# Detection and viral RNA shedding of SARS-CoV-2 in respiratory specimens relative to symptom onset among COVID-19 patients in Bavaria, Germany − Addendum

**DOI:** 10.1017/S095026882100159X

**Published:** 2021-07-26

**Authors:** Tom Woudenberg, Ute Eberle, Durdica Marosevic, Bernhard Liebl, Nikolaus Ackermann, Katharina Katz, Andreas Sing

**Affiliations:** 1 Unit of Infectious Diseases Epidemiology, Bavarian Health and Food Safety Authority, Oberschleissheim, Germany; 2 ECDC Fellowship Programme, Field Epidemiology path (EPIET), European Centre for Disease Prevention and Control (ECDC), Stockholm, Sweden; 3 Unit of Virology, Bavarian Health and Food Safety Authority, Oberschleißheim, Germany; 4 State Institute of Health, Bavarian Health and Food Safety Authority, Oberschleißheim, Germany; 5 Ludwig Maximilians-Universität, Munich, Germany; 6 Unit of Public Health Microbiology, Bavarian Health and Food Safety Authority, Oberschleißheim, Germany

The original publication of this article contains an error on Page 13.

The current phrase reads:

However, we expect that lower Ct-values are associated with a lower probability of isolating virus in culture [28–32].

The phrase should be:

‘However, we expect that higher Ct-values are associated with a lower probability of isolating virus in culture [28–32].’
